# Therapeutic potential of PPARα agonist in ligature-induced experimental periodontitis

**DOI:** 10.1590/1678-7757-2021-0648

**Published:** 2022-03-11

**Authors:** Ying Chen, Yang Hu

**Affiliations:** 1 Usak University The Forsyth Institute Department of Immunology and Infectious Diseases Cambridge Massachusetts USA The Forsyth Institute, Department of Immunology and Infectious Diseases, Cambridge, Massachusetts, USA.

**Keywords:** Periodontitis, Fenofibrate, Inflammation, Bone loss

## Abstract

Inflammation-related immune responses and bone metabolism lead to extensive tooth loss in periodontitis.

**Objective:**

This study aims to investigate the effect of peroxisome proliferator-activated receptor (PPAR) alpha agonist anti-inflammatory treatment *in vitro* and in ligature-induced experimental periodontitis *in vivo* .

**Methodology:**

Splenocytes were isolated from C57BL/6J mice and cultured for 48 hours under the following conditions: control, P. gingivalis lipopolysaccharide (LPS) (1 µg/ml); experimental, LPS (1 µg/ml) + PPARα agonist (fenofibrate) at 1, 10, 50, 100 µM. MRNA and secreted protein levels of TNF-α expression were detected by RT-qPCR and ELISA, respectively. Silk ligatures (7-0) were tied around maxillary second molars of C57BL/6J mice for two weeks. Optimized doses of fenofibrate (50 µM) and vehicle control were injected into the contralateral side of the palatal gingiva on days three, six, and nine. At day 14, bone resorption, osteoclastogenesis, and gingival mRNA expression levels of TNF-α, IL-1β, IL-6, and RANKL/OPG were measured by micro-computed tomography, Tartrate-resistant acid phosphatase (TRAP) staining, and Real-time quantitative PCR, respectively.

**Results:**

TNF-α expression in cultured spleen cells were significantly increased in the presence of LPS, when compared with the control group, and significantly reduced by fenofibrate treatment in a dose-dependent manner from 1-100 µM (p<0.05). Gingival mRNA levels of TNF-α, IL-1β, IL-6, and the ratio of RANKL/OPG, were significantly decreased after injection of fenofibrate, when compared to the control side (p<0.05). Periodontal bone loss and TRAP positive cell formation were significantly decreased on the side with an injection of fenofibrate, as compared to the control side (p<0.05).

**Conclusions:**

An anti-inflammatory treatment, PPARα agonist, inhibited inflammation and periodontal bone loss in ligature-induced experimental periodontitis.

## Introduction

The periodontium consists of the gingiva, periodontal ligament, cementum, and the alveolar bone proper. These teeth-surrounding tissues support teeth in the maxillary and mandibular bones and maintain teeth functioning.^1^ Periodontal disease is a progressive inflammatory process affecting teeth-surrounding tissues, destroying bone-supporting tissues, inducing bone resorption, and constituting the leading cause of bone loss in teeth.^2^

A various range of inflammatory cytokines and signaling pathways have been shown to mediate the pathological process of periodontitis.^2^ Among these, inflammatory factors, tumor necrosis factor-alpha (TNF-α), and interleukin-I (IL-1) play the causative pathological roles of destroying periodontal tissues.^3^ The excessive production of IL-1 and TNF is believed to be an overreaction of the host’s immune response to periodontal pathogens.^4^ IL-1 induces adhesion molecules and a number of inflammatory factors to attack the periodontal tissues, resulting in the loss of connective tissue attachment, osteoclast formation, bone resorption, and loss of alveolar bone.^5^ On the other hand, TNF-α mediates the loss of fibroblasts in the pathogenic process of periodontal infections.^5 – 7^ Antagonizing IL-1 and TNF-α in experimental periodontitis has shown the beneficial effects of inhibiting the activity of pro-inflammatory cytokines and the further spread of the inflammation in the periodontal tissues.^7^

PPARs are members of the nuclear hormone receptor and function as transcription factors regulating the expression of genes in the metabolism and inflammation.^8^ Binding with selective ligands activates PPAR and results in heterodimerization with the retinoid X receptor (RXR), which regulates gene expression^8^ . A previous study has shown that PPARα activation upregulates the overexpression of the IL-1 receptor antagonist (IL-1ra).^9^ It has also been shown that activated PPARα binds to c-Jun and to the p65 subunit of NF-κB,^10 , 11^ thereby inhibiting NF-κB mediated signaling, including its downstream factors, such as TNF-α. Different PPAR subtypes show distinct tissue distributions.^12^ Studies have shown that both PPARα and RXR are expressed in the gingival tissue,^13^ and that PPARα expression is higher in periodontitis and peri-implantitis groups than in healthy patients, whereas RXR shows a reverse pattern to PPARα, higher in healthy individuals than in periodontitis and peri-implantitis patients.^14^

In this study, we evaluate the anti-inflammatory effect of fenofibrate, a PPARα agonist, in an experimental ligature-induced periodontitis model. Our study focuses on key inflammation events, including the effect of fenofibrate on the production of IL-1 and TNF-α, and bone loss.

## Methodology

Animal: Wild-type (WT) C57BL/6 mice (Jackson Laboratory) aged eight to 10 weeks were used for experiments. Half of the mice used were males and half, females. A total of 24 mice (six groups, four per group) was used in an *in vitro* study and a total of 24 mice (two groups, 12 per group) was used in an *in vivo* study. The animal experiments were approved by the Institutional Animal Care and Use Committee.

Cell preparation and culture: Mice spleens were collected and carefully ground into an IMDM medium (Gibco) through a 2 cm X 2 cm metal mesh. After going through a 100 μm cell strainer, the ACK lysis buffer (Life Technologies) was applied to lysis erythrocytes. After, a 40-μm cell strainer was used. Then, splenocytes (5 × 10^6^ cells/well) were cultured in 96-well tissue culture plates with 200 µl of an IMDM medium containing 10% FBS, 2 mM L-glutamine, 100 U/mL penicillin, 100 U/mL streptomycin, 2.5μg/mL Fungizone, and 0.1% 2-ME for 48h with the following conditions as control: *P. gingivalis* lipopolysaccharide (LPS) (1 µg/ml), LPS(1 µg/ml) + fenofibrate (1 µM), LPS(1 µg/ml) + fenofibrate (10 µM), LPS(1 µg/ml) + fenofibrate (50 µM), and LPS(1 µg/ml) + fenofibrate (100µM).

Animal model and local administration: Experimental periodontitis was induced in mice with a silk (7-0, Fisher Scientific) ligature around maxillary second molars for 14 days, as previously described.^15^ All mice were randomly distributed into two groups. For the *in vivo* study, a power analysis was performed with the JMP Pro 13 statistical software based on preliminary data, α set to 0.05, and β, to 0.2 (which allows for a 0.8 power) for the primary outcome of bone loss, TRAP staining, and gingival mRNA expressions. Group 1 (n=12 animals/group) had ligatures only on the left maxillary second molar and no ligature on the right side. Group 2 (n=12 animals/group) had ligature on both maxillary second molars, with the left side injected with fenofibrate (50 µM, 2 µl) and the right side with Vehicle (PBS, 2 µl). On day three, six, and nine of the ligature, 2 µl of fenofibrate or PBS were administrated into the palatal gingival papilla around the second molar of mice. On day 14, all mice were euthanized by CO_2_ inhalation. During the ligature period, if ligatures fell off from the maxillary molar, they were immediately replaced at the daily check.

ELISA: The Mouse TNF-α ELISA MAX^™^ Standard kit (Biolegend) was used to test secreted TNF-α protein levels in the supernatant of cultured splenocytes. A standard curve was performed with each assay which included duplication of each sample with absorbance at 450 nm via a microplate reader (BioTek). According to the manufacturer’s protocol, the TNF-α concentration (ng/mL) of each sample was computed from the standard curve.

**Figure 1:**
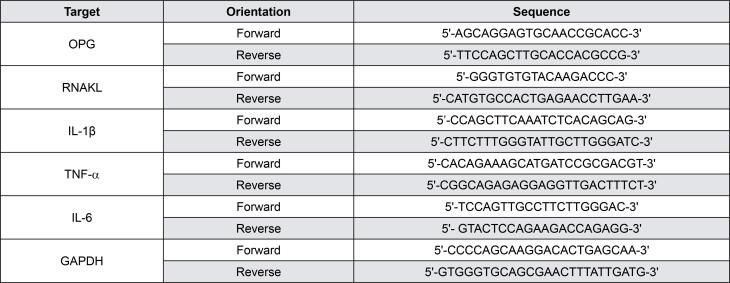
Primer sequences used for real-time PCR

Real-time PCR: Palatal gingival tissues were harvested from around the maxillary second molar and homogenized via a tissue homogenizer. The PureLink^®^ RNA Mini Kit (Ambion) was used to extract the total RNA from cultured splenocytes or homogenized gingival tissues. CDNA was synthesized with the SuperScript II Reverse Transcriptase kit (Invitrogen). The mRNA expression of TNF-α, IL-1β, IL-6, RANKL, and the OPG of samples was measured by real-time qPCR via the LightCycler^®^ 480 Instrument system (Roche), as previously described.^15^ All primer sequences are shown in Figure 1 .

Bone morphometric analysis: Mice skulls were harvested and defleshed by beetle colonies. A high-resolution scanner (mCT-40, Scanco Medical, Sweden) was used to analyze these skulls. The Seg3D software was used to establish the quantitative 3-D images, followed by measurements of bone resorption volume, as previously described,^15^ with blinding of groups. Briefly, the volume of interest (VOI) of a cylinder with a diameter of 1.0 mm and a height of 1.0 mm was defined from the cement–enamel junction plane. The empty space volume (ESV) surrounding the teeth was measured by the total VOI volume minus bone volume. Then, the bone loss of each sample was calculated by its ESV minus the average ESV in the no ligature control group.

Tissue histological analysis: The maxillary part of skulls was harvested and put in 4% formaldehyde for fixation. Then, skulls were decalcified in 10% EDTA for two weeks at 4ºC, with sloshing, and embedded in paraffin. Five μm tissue sections were cut in parallel with the long axis of the molars. These sections were stained with an acid phosphatase kit (378A, Sigma) and counterstained with hematoxylin for tartrate-resistant acid phosphatase (TRAP) analysis. The number of multinucleated TRAP-positive cells which were considered osteoclasts were counted (n=6 animals/group) as previously described.^15^

### Statistical analysis

Quantitative data were expressed as means ± SD. Statistical analysis was performed using unpaired Student’s *t* -test for comparisons of any two groups of data sets. Statistical significance was set at p<0.05.

## Results

### Effects of PPARα agonist treatment on TNF-α mRNA level of splenocytes induced by LPS.

**Figure 2:**
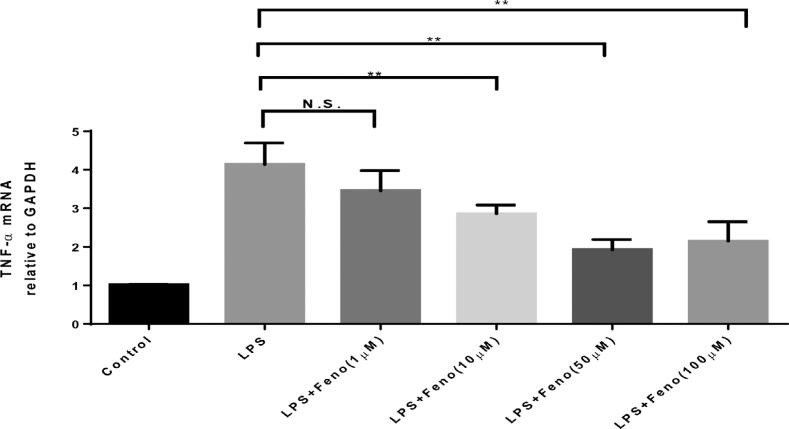
Effects of differentd oses of PPARα A treatment on TNF-α mRNA level of splenocytes induced by LPS Mouse splenocytes were separated from C57/BL6 wild-type (WT) mice aged eight to 10 weeks and treated with LPS (1 µg/ml), LPS (1 µg/ml) + Fenofibrate (1 μM, 10 μM, 50 μM, and 100 μM) for 48 hours. The mRNA levels of TNF-α were measured and analyzed via qRT-PCR. (mean ± SD, n=4, compared with LPS group, *p<0.05, **p<0.01, N.S., no significance)

TNF-α is a proinflammatory cytokine released by macrophages which plays a substantial pathologic role in periodontitis.^3^ TNF-α specially mediates the loss of fibroblasts and is associated with bone loss in periodontitis. We, therefore, tested the ability of fenofibrate to reduce the production of TNF-α. The splenocytes collected from mice were incubated with lipopolysaccharide (LPS) (10 ng/ml), one of the most potent stimuli for macrophages to produce large quantities of proinflammatory cytokines, including TNF-α. As Figure 2 shows, LPS induced a four-fold increase in the expression of TNF-_α_ at a transcriptional level, which is significantly reduced by fenofibrate, in a dose-dependent manner, from 1 to 100 µM.

### Effects of PPARα agonist treatment on TNF-α protein expression level of splenocytes induced by LPS.

**Figure 3:**
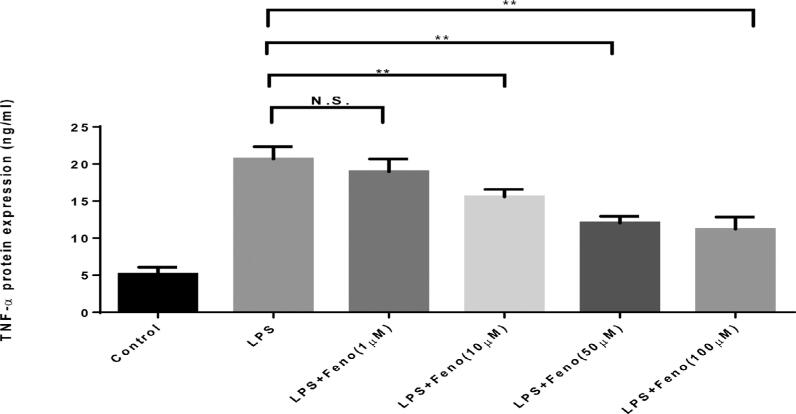
Effects of different doses of PPARα A treatment on TNF-α protein expression level of splenocytes induced by LPS Mouse splenocytes were separated from C57/BL6 wild-type (WT) mice aged eight to 10 weeks and treated with LPS (1 µg/ml), LPS (1 µg/ml) + Fenofibrate (1 μM, 10 μM, 50 μM, and 100 μM) for 48 hours. The protein expression levels of TNF-α were measured by ELISA kits and analyzed. (mean ± SD, n=4, compared with LPS group, *p<0.05, **p<0.01, N.S., no significance)

Next, we tested the direct effect of fenofibrate on the expression of TNF-α induced by LPS at a protein level in mice splenocytes. At protein levels, LPS induced a robust elevation of TNF-α, to a level over 20-fold that of control untreated cells ( Figure 3 ). Treatment with fenofibrate significantly decreases the elevation of TNF-α, in a dose-dependent manner. Notably, based on both TNF-α mRNA and protein levels changes above, a 50 µM dose of fenofibrate achieved the maximal inhibition of the LPS-induced increase of TNF-α. Thus, this dose was considered as an optimized dose for an *in vivo* study.

### PPARα agonist inhibited bone loss in a ligature-induced experimental periodontitis mouse model.

**Figure 4:**
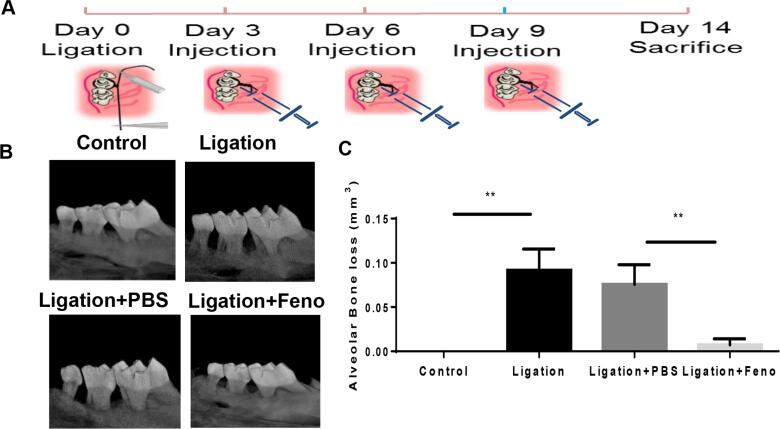
PPARαA inhibited bone loss in a ligature-induced experimental periodontitis mouse model Silk ligatures were tied around maxillary second molars on both sides of C57/BL6 mice mouths on day 0; fenofibrate (50 µM, 2 µl) or Vehicle (PBS, 2 µl) was injected on days three, six, and nine (A). Maxilla were collected on day 14, measured by 3D micro-CT (B), and analyzed as bone resorption (volume/mm3¬) (C). (mean ± SD, n=6 mice per group, **p<0.01).

To explore the protective potential of fenofibrate on periodontitis *in vivo* , we first tested the effect of fenofibrate on bone loss in a ligature-induced experimental periodontitis mouse model ( Figure 4A ). We observed that the application of ligatures resulted in dramatically increased levels of alveolar bone destruction, and mice treated with fenofibrate exhibited a significant reduction in alveolar bone loss to a level comparable to the control, as indicated by volume measurements of bone loss ( Figure 4B , 4C ).

### PPARα agonist reduced TRAP-positive cells in a ligature-induced experimental periodontitis mouse model.

**Figure 5:**
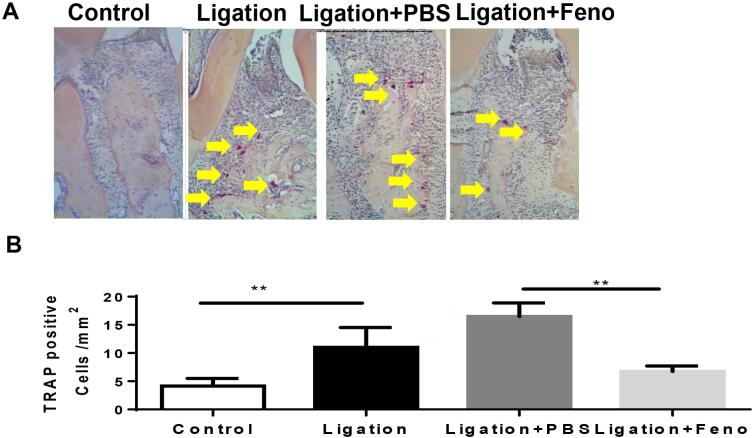
PPARα A reduced TRAP-positive cells in experimental periodontitis (A) TRAP staining was performed on tissue sections from all mice groups and images of periodontal tissues were analyzed at 200 X magnification. (B) Bar chart analysis showed the number of multinucleated TRAP+ cells along the alveolar bone surface. (means ± SD (/mm2, n=6). *, p< 0.05, **, p< 0.01).

We hypothesized that the observed reduction in alveolar bone effects was associated with the amelioration of bone resorption by the osteoclasts. To test this hypothesis, we evaluated the effects of fenofibrate on TRAP activity, a valid cytochemical marker for the identification of osteoclasts in the experimental periodontitis model. As shown in Figure 5 , ligatures induced significant TRAP-positive staining and a corresponding statistical increase of TRAP-positive cells into periodontal tissues. Strikingly, mice treated with fenofibrate exhibited a marked decrease in TRAP-positive cell levels to an almost complete reversal. Our study confirms that fenofibrate ameliorates bone loss and reduces alveolar bone resorption in periodontal disease.

### PPARα agonist decreased gingival mRNA levels of IL-1, IL-6, TNF-α, and RANKL/OPG in ligatures-induced experimental periodontitis.

**Figure 6:**
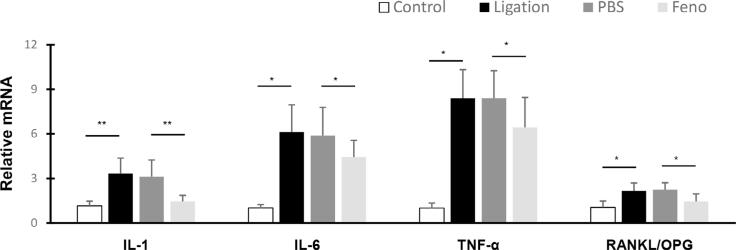
PPARα A treatment decreased gingival mRNA levels of IL-1, IL-6, TNF-α, and RANKL/OPG in ligature-induced experimental periodontitis Silk ligatures were tied around maxillary second molars of both sides of C57/BL6 mice mouths on day 0; fenofibrate (50 µM, 2 µl) or Vehicle (PBS, 2 µl) was injected on days three, six, and nine. Gingival tissue mRNA levels of TNF-α, IL-1, IL-6, and ratio of RANKL/OPG were measured and analyzed. (mean ± SD, n=6, *p<0.05, **p<0.01).

Fenofibrate amelioration reduced alveolar bone resorption in the ligature-induced experimental periodontitis model described above. To further explore the underlying molecular mechanism responsible for disease amelioration by fenofibrate, we examined the changes in the expression of cytokines and osteogenic markers in periodontal tissues at the transcriptional level. Our study showed that mice with ligature-induced periodontitis exhibited a significant upregulation of IL-1, IL6, and TNF-α expression, which were downregulated by the administration of fenofibrate, compared with control experimental periodontal disease mice ( Figure 6 ). We have also examined the effect of fenofibrate on the ratio of RANKL, a ligand important for activating osteoclastogenesis and responsible for bone resorption,^15^ and RANKL extracellular inhibitor osteoprotegerin (OPG)^16 , 17^ at transcriptional levels in the experimental models. Our study showed that fenofibrate administration significantly downregulates the increase in the ratio of RANKL/OPG in periodontal disease mice ( Figure 6 ).

## Discussion

In our ligature-induced experimental periodontitis model, the levels of TNF-α and IL-1 were elevated, which is in line with the findings in patients with periodontal diseases.^5^ In patients with chronic periodontitis, aggressive periodontitis, and peri-implantitis, the levels of TNF-α and IL-1 were elevated and contributed to patients’ susceptibility to the disease.^16 – 18^ Graves’ study showed that, when TNF-α and IL-1 were antagonized with antibodies, a cause-and-effect relationship between inflammation degree and periodontitis severity was verified.^5^

Our study showed that increases in TNF-α, IL-1, and IL6 were reduced by fenofibrate administration in the ligature-induced experimental periodontitis model. In *in vitro* studies, fenofibrate decreases TNF-α expression and mRNA levels, in a dose-dependent manner, , suggesting that fenofibrate regulates the expression of these pro-inflammatory factors by interfering with their transcription.

Fenofibrate is an agonist of PPARα, a nuclear transcriptional factor.^8^ Schaefer’s study showed that LPS increases TNF-_α_ in both wild-type and PPARα-null mice. Treatment with PPARα agonist reduces TNF-α in wild-type mice but not in PPARα-knockout mice,^19^ suggesting that PPARα is essential for fenofibrate lowering TNF-α.

PPARα regulates gene transcription by forming a transcriptional complex with RXR. Andriankaja, et al.^20^ (2012) reported that both PPARα and RXR were expressed in periodontal tissues; PPARα levels were low in ligature-induced periodontitis rodents but was three times higher after ligatures were removed and the periodontitis solved. Unlike PPARα alteration, the level of RXR is unaffected by periodontitis induction,^20^ suggesting that PPARα is a potentially therapeutic target for periodontitis. In this study, we have been unable to determine how TNF-α and IL-6 are regulated by PPARα in periodontitis, i.e., whether via the PPAR response element (PPRE) or via other PPARα target genes.

Another PPARα target gene worth mentioning is NF-kB. NF-kB is a key transcriptional factor required for TNF-α and IL6 induction.^21 , 22^ PPARα downregulates NF-kB by binding to c-Jun and to the p65 subunit of NF-kB,^10^ subsequently downregulating the transcription of TNF and IL6.^23^ On the other hand, the initiation of the NF-kB-IL6 signaling requires the binding of TNF-α to its receptor.^24 , 25^ It is unclear whether the anti-inflammatory effects of fenofibrate are due to the synergistic negative effects of PPAR on NF-kB and TNF-α.

Both TNFα and IL-1 play bone destructive roles which contribute to bone loss in periodontitis. For example, Algate’s, as many other studies, showed TNF-α playing a potent catabolic role by stimulating osteoclastic bone resorption and the suppression of osteoblastic bone formation.^6^ In a study of a periodontitis model, Apolinario and Pereira showed that IL-1 contributed to alveolar bone resorption and attachment loss, which was reduced by inhibiting IL-1 activity.^26 , 27^

In our study, fenofibrate exhibited a preventative effect on the deterioration of the alveolar bone in the ligature-induced periodontitis model. Fenofibrate prevented bone loss, as the alveolar bone cells were maintained at a level comparable to non-periodontitis control mice. Fenofibrate also inhibited bone resorption, as reflected in significantly lower TRAP-positive cell levels, an indicator of osteoclast activity, and significantly lower RANKL/OPG levels than controls. Okamoto’s study showed that fenofibrate suppresses osteoclast differentiation by inhibiting the NF-kB signaling pathway.^11^ Stunes’s studies of PPARs and bone structure in ovariectomized rats showed that activation of PPARγ was associated with bone loss, whereas activation of PPARα with fenofibrate showed a positive bone protective effect.^28 , 29^ However, in this study, we were unable to determine if this protective effect of fenofibrate on the alveolar bone is due to a direct PPARα effect on bone cells or to an indirect inflammation inhibition effect. A further investigation will be warranted to explore this possibility.

Ligature duration (14 days), on our experimental periodontitis mice model, is one of the limitations of our study. Although our previous studies showed that 14-day ligatures were enough to generate significant bone loss and changes in inflammatory cytokines,^15 , 30^ it is very important for us to study these changes in shorter (7 days) or longer (21 days, 28 days or more) periods. Based on the literature on mice ligature-induced experimental periodontitis models,^31^ progressive alveolar bone resorption develops into an acute (0-14 days, pronounced inflammation and alveolar bone loss) and chronic phases (14-21 days, no significant progression of bone loss). Thus, similar future studies need to address the mice ligature-induced experimental periodontitis model to address this limitation.

Inflammation plays a crucial pathogenic role in the process of periodontitis. This study, investigating the effect of fenofibrate, a PPARα agonist, on inflammatory response and bone protection in a ligature-induced experimental periodontitis model, aimed to explore a new therapeutic target as an alternative strategy for periodontitis. In summary, our study has provided first-hand evidence that fenofibrate has a potentially therapeutic benefit for treating periodontitis.
